# The association between ambient temperature and elite racewalking performance in the olympics and world championships

**DOI:** 10.3389/fspor.2025.1681100

**Published:** 2025-11-07

**Authors:** Xiangning Zhang, Dandan Cui, Zili Jiang, Wenchao Yang

**Affiliations:** 1 Institute of Artificial Intelligence in Sports, Capital University of Physical Education and Sports, Beijing, China; 2 China Institute of Sport Science, Beijing, China; 3 Chinese Athletics Association, Beijing, China

**Keywords:** race segments, performance levels, elite athlete, racewalking, athletics

## Abstract

**Introduction:**

Global warming has become one of the major challenges in athletics, with evidence suggesting that racewalking is susceptible to high ambient temperature, though the magnitude of this relationship remains uncertain. This study aimed to investigate the relationship between weather conditions and racewalking performance in major international events, and examine differences across sex, performance levels, and race segments.

**Methods:**

Data on results, ambient temperatures, and relative humidity were collected from eight events over ten years for women's and men's 20 km and men's 50 km racewalking. Statistical analysis included the coefficient of variation (CV) for performance stability and Welch's ANOVA for event comparisons. Correlation analysis was used to examine the relationship between weather conditions and performance, while Generalized Linear Models (GLMs) identified key variables associated with performance across various factors.

**Results:**

The women's 20 km had the highest performance stability (CV = 4.89%); other disciplines were also stable (CV < 10%). Ambient temperature significantly correlated with finishing times (*r* = 0.38–0.92, *p* < .05), but relative humidity showed minimal correlation (*r* = −0.48, *p* < .05). GLMs showed ambient temperature was associated with performance, with varying sensitivity across events: finishing time increased by +1.15%/°C, +0.96%/°C, and +0.77%/°C per degree t rise in temperature, respectively. The top8 athletes in women's 20 km had stronger associations (+0.64 to +0.67% vs. +0.42%), while athletes below 4th in men's 20 km showed higher sensitivity (+0.74% to +1.33%). All athletes in 50 km exhibited pronounced trends (start: −1.44 to −3.14%; end: +2.43 to +4.64%). The model showed a stronger association between ambient temperature and racewalking performance in the first half (pseudo *R*^2^ = 0.30–0.74 vs. 0.11–0.28).

**Conclusion:**

The racewalking performance of elite athletes shows correlation with ambient temperatures, especially in the first half, with these associations varying by sex, performance levels, and race segments. These findings underscore the necessity for athletes and coaches to develop individualized, segment-specific pacing and thermal management strategies to optimize performance in thermally challenging conditions.

## 1 Introduction

As climate change increases the frequency of extreme heat events (1), major international events are increasingly being held under elevated ambient temperatures (2). High environmental heat has thus become a critical factor affecting athletic performance. Strategies to address these challenges, such as venue relocation (3), event cancellations, and the development of heat response strategies (4, 5), have attracted growing attention (2, 6, 7). Prolonged exposure to heat poses a risk of thermoregulation failure for athletes (8), with endurance athletes particularly susceptible (1, 9). Studies indicate that marathon performance declines in high ambient temperatures (10, 11), and the world record may stagnate or slow in the future due to thermal constraints. Additionally, the interaction between ambient temperatures and marathon performance is influenced by factors such as sex (12), performance levels (10), and race segments (13). However, most research on marathons under high ambient temperatures (excluding the Olympics and World Championships) tends to overlook the distinction among runners in different performance levels. Moreover, the majority of these mass-participation marathons are strategically scheduled in the spring or autumn to avoid the hottest months. This conflation of runner abilities, combined with the typically milder ambient temperature, may lead to different impact patterns compared to studies focusing on elite endurance athletes competing in major international events, which are often held in more severe heat conditions.

Racewalking, like the marathon, is a long-distance endurance discipline that involves diverse performance-influencing factors. However, compared to marathon running, it has received relatively limited scientific attention, particularly concerning the impact of environmental factors such as ambient temperature on the performance of its predominantly elite participants. Technically, it possesses distinct physiological requirements (14) and pronounced biomechanical specificity (15) compared to other endurance disciplines. Additionally, athletes often struggle to maintain a consistent pace at racewalking duration, with only 13.8% of athletes adhering to their pre-programmed pace (3). This difficulty in maintaining pace suggests that external factors, such as weather conditions, may influence the pacing strategies of elite athletes in racewalking. However, systematic research on such an impact is currently lacking, especially regarding sex, performance levels, and race segments during major international events.

Emerging evidence indicates that racewalking athletes may face greater heat stress risks compared to other athletes (14, 16, 17), and are more susceptible to heat illness than those in similar running endurance disciplines (14). Mantzios et al. found that most heat illness cases during the World Championships occurred in racewalking (17). Therefore, further understanding of the relationship between ambient temperature and racewalking performance is essential to develop effective thermal management strategies at racewalking duration, to safeguard athlete health and optimize performance.

This study aims to analyze the relationships between ambient temperatures and racewalking performance through a systematic, multi-level analysis that considers the influence of key interacting factors, including sex, performance levels, and race segments. First, we will investigate whether weather conditions have a correlation with racewalking performance. Second, we will examine how the relationship between ambient temperature and racewalking performance varies by sex, performance levels, and race segments, providing insights for future race strategies among elite athletes.

## 2 Methods

### 2.1 Data sources and variable definition

Data were collected from eight major international racewalking events held between 2015 and 2024, including the Olympic Games (OG) and the World Championships (WC) organized by the World Athletics. Data were primarily obtained from the official websites of the World Athletics and the International Olympic Committee (IOC), which provide authoritative records of both competition results and meteorological conditions. A preliminary assessment conducted prior to data collection revealed substantial inconsistencies in climate data across third-party platforms, whereas the official datasets from the World Athletics and IOC are athletic-specific, standardized, and therefore more appropriate for performance analysis in athletics. For events with missing data, supplementary information was retrieved from official event footage, IOC replays, and the WeatherSpark platform, whose historical weather data showed relatively greater consistency with official records compared to other non-official sources.

To ensure data consistency and accuracy, a multi-step standardization procedure was applied. This included: (1) converting all performance times (finishing, first half, and second half) into a single unit (total seconds) for analysis; (2) excluding all records of athletes who were disqualified (DSQ) or did not finish (DNF) to make the dataset valid.

The study covers three disciplines: women's 20 km, men's 20 km, and men's 50 km, including eight major international athletics events held over the past decade: the Beijing 2015 (WC), the Rio 2016 (OG), the London 2016 (WC), the Doha 2019 (WC), the Tokyo 2020 (OG), the Oregon 2022 (WC), the Budapest 2023 (WC), and the Paris 2024 (OG). Given that the data are publicly available, no written and informed consent from individual athletes was required.

Weather conditions are assessed using the ambient temperatures (starting and ending) and relative humidity. Due to the absence of other weather variables from official race organizers' records, only these two indicators were included to ensure data reliability. Racewalking performance was measured using racewalking results (i.e., start-to-finish time per unit distance), where quicker times indicate better results. The metrics analyzed included finishing time, first half time, and second half time. Performance levels were further categorized into three tiers based on final race rankings: Medalists, Rank 4–8, and Rank > 8.

### 2.2 Statistical analysis

To assess the stability of performance across the three disciplines over the past decade, the CV was applied to quantify random errors from variability in athlete participation. A CV below 10% indicates high performance stability with low variability (18). To ensure appropriate statistical modeling, the distribution characteristics of finishing times were evaluated through Shapiro–Wilk normality tests and skewness and kurtosis analysis (thresholds: |skewness|>1, |kurtosis-3|>1), with gamma parameters estimated via maximum likelihood. Nonparametric methods were automatically triggered when thresholds were exceeded. Potential outliers were screened for using box plots and the interquartile range (IQR) method and were subsequently examined for legitimacy.

Based on the outcomes of these distributional tests, robust statistical methods were selected. To analyze differences in finishing times between events, Welch's ANOVA was chosen due to its effectiveness in cases with unequal variances. Spearman's rank correlation analysis was then conducted to explore the relationships between weather conditions and racewalking performance. This method is robust to non-normal data distributions and the presence of outliers. This analysis was used to assess how ambient temperatures and relative humidity relate to performance, stratified by race segments and performance levels. Specifically, the analysis examined the correlation between relative humidity and starting temperature and both first and second half times, as well as between ending temperature and second half time—consistent with race chronology. Correlation strength was interpreted using conventional medical field standards: 0.1 < |*r*| < 0.3 indicates poor; 0.3 < |*r*| < 0.5 indicates weak; 0.5 < |*r*| < 0.7 indicates moderate; 0.7 < |*r*| < 1 indicates strong (19).

To further analyze the associations between ambient temperatures and racewalking performance, a Generalized Linear Model (GLM) was constructed using a gamma distribution and a log link function, suitable for non-negative, right-skewed response variables. The model incorporated both starting and ending temperatures as independent variables, with finishing time, first half time, and second half time as dependent variables. Performance levels were included as a stratification variable to assess potential differences in the association between ambient temperature and racewalking performance. To ensure symmetry, the model also assessed the influence of ending temperature on the first half time. Both overall and stratified models were developed to provide a more comprehensive analysis. A segment-based approach was used to evaluate associations between ambient temperature and 10-km racewalking splits. The model's goodness-of-fit is assessed using McFadden's pseudo-*R*^2^, calculated as Equation 1:

$$\mathrm{Pseudo}-R^{2}=1-\frac{D_{1}}{D_{0}}$$ (1)

Where D₁ is the deviance of the fitted model, and D₀ is the deviance of the null model. The value range of this statistic is from 0 to 1, with larger values indicating a better fit of the model (20). Although typically lower than traditional R^2^ values, scores between 0.2 and 0.4 are considered a very good model for GLMs due to model complexity and the influence of non-normal distributions on log-likelihood estimates (21, 22).

To interpret the practical significance of model coefficients, log-transformed estimates from both overall and stratified models were converted into percentage and absolute time changes. The percentage change in finishing time per 1°C increase in ambient temperature was calculated using the formula 2:

$$\mathrm{Percentage}\;\mathrm{Change}=(e^{\beta }-1)\times 100\text{\%}$$ (2)

Absolute changes were derived by multiplying this value by the average finishing time for each race using the formula 3:

$$\mathrm{Absolute}\;\mathrm{Time}\;\mathrm{Change}=\mathrm{Average}\;\mathrm{Finishing}\;\mathrm{Time}\times (e^{\beta }-1)$$ (3)

where *β* represents the model coefficient.

For stratified analyses, absolute time changes were estimated separately for each race segments. To assess the combined impact of starting and ending temperatures, the following formulas were used as formulas 4–6:

$$\mathrm{Combined}\;\mathrm{Regression}\;\mathrm{Coefficient}=\beta_{\mathrm{start}}+\beta_{\mathrm{end}}$$ (4)

$$\mathrm{Combined}\;\mathrm{Percentage}\;\mathrm{Change}=(e^{\beta_{\mathrm{start}}+\beta_{\mathrm{end}}}-1)\times 100\text{\%}$$ (5)

$$\mathrm{Combined}\;\mathrm{Absolute}\;\mathrm{Time}\;\mathrm{Change}=\mathrm{Average}\;\mathrm{Finishing}\;\mathrm{Time}\times (e^{\beta_{\mathrm{start}}+\beta_{\mathrm{end}}}-1)$$ (6)

To visualize estimated association magnitudes, forest plots were used to display standardized regression coefficients with 95% confidence intervals (consistent across all estimates). Using performance levels as stratification variables, the plots presented segment-based analyses. This approach facilitated a direct comparison of temperature associations with performance across subgroups while identifying statistically significant associations. All analyses and visualizations were conducted using Python 3.8 in the PyCharm IDE, and two-sided *p*-values < 0.05 were considered statistically significant.

## 3 Results

### 3.1 Descriptive analysis

Descriptive statistics and contextual details for finishing times across all events are summarized in Table 1, including event location, local start and end times, number of finishers (*N*), and mean finishing times with standard deviations (SD). Performance stability was high across all disciplines. The women's 20 km exhibited the lowest variability (CV = 4.89%), followed by the men's 20 km (5.95%) and the men's 50 km (6.28%). A Welch's ANOVA confirmed statistically significant differences in finishing times across events within each discipline (all *p* < .001).

**Table 1:** Contextual information and descriptive statistics of finishing times for each competition.

Discipline	Location	Local start time	Local end time	*N*	Mean ± SD (s)	CV	Welch *F*-value
Women's 20 km	Beijing 2015 WC	8:30:00	10:11:00	41	5,633.66 ± 204.20	4.89%	15.24**
	Rio 2016 OG	14:30:00	16:15:06	63	5,742.92 ± 259.63		
	London 2017 WC	12:21:00	14:03:00	48	5,555.10 ± 203.21		
	Doha 2019 WC	23:58:00	1:48:00	39	5,969.10 ± 250.69		
	Tokyo 2020 OG	16:30:00	18:17:19	53	5,781.68 ± 282.47		
	Oregon 2022 WC	13:09:00	14:55:00	36	5,629.83 ± 239.73		
	Budapest 2023 WC	7:15:00	8:59:00	40	5,546.75 ± 227.44		
	Paris 2024 OG	9:50:00	11:37:26	43	5,532.44 ± 257.95		
Men's 20 km	Beijing 2015 WC	8:30:00	10:07:00	50	5,052.60 ± 163.48	5.95%	31.48**
	Rio 2016 OG	14:30:00	16:03:58	63	5,041.76 ± 200.46		
	London 2017 WC	14:19:00	15:51:00	58	4,983.03 ± 185.19		
	Doha 2019 WC	23:29:00	1:25:00	40	5,652.80 ± 308.64		
	Tokyo 2020 OG	16:30:00	18:09:38	52	5,201.87 ± 247.84		
	Oregon 2022 WC	15:09:00	16:56:00	43	5,122.63 ± 305.19		
	Budapest 2023 WC	10:49:00	12:22:00	47	4,920.21 ± 199.41		
	Paris 2024 OG	8:00:00	9:29:14	46	4,927.80 ± 155.53		
Men's 50 km	Beijing 2015 WC	7:30:00	12:03:00	39	14,149.44 ± 644.30	6.29%	27.51**
	Rio 2016 OG	8:00:00	12:39:48	49	14,438.59 ± 866.56		
	London 2017 WC	7:46:00	12:07:00	33	13,765.74 ± 414.06		
	Doha 2019 WC	23:30:00	4:35:00	28	15,714.36 ± 814.37		
	Tokyo 2020 OG	5:30:00	11:23:09	47	14,656.68 ± 733.22		

** *P* < .001.

The Shapiro–Wilk test revealed significant deviations from normality across all events (women's 20 km *W* = 0.98, *p* < .001; men's 20 km *W* = 0.90, *p* < .001; men's 50 km *W* = 0.94, *p* < .001). All distributions were right-skewed (skewness: women's 20 km = 0.47; men's 20 km = 1.34; men's 50 km = 0.89). Furthermore, their tail characteristics varied compared to a normal distribution (baseline kurtosis = 3), with the men's events being leptokurtic (kurtosis: men's 20 km = 5.12; men's 50 km = 3.52) while the women's event was platykurtic (kurtosis = 2.83). Outlier analysis (Supplementary Figure S1) identified no potential outliers in the Women's 20 km event but found 14 in the Men's 20 km and 4 in the Men's 50 km data; these were retained as valid data points. Further examination, these were confirmed to be legitimate but unusually slow finishing times. To maintain the integrity of the dataset and reflect actual competition conditions, all data points were retained for subsequent analysis.

**Figure 1:**
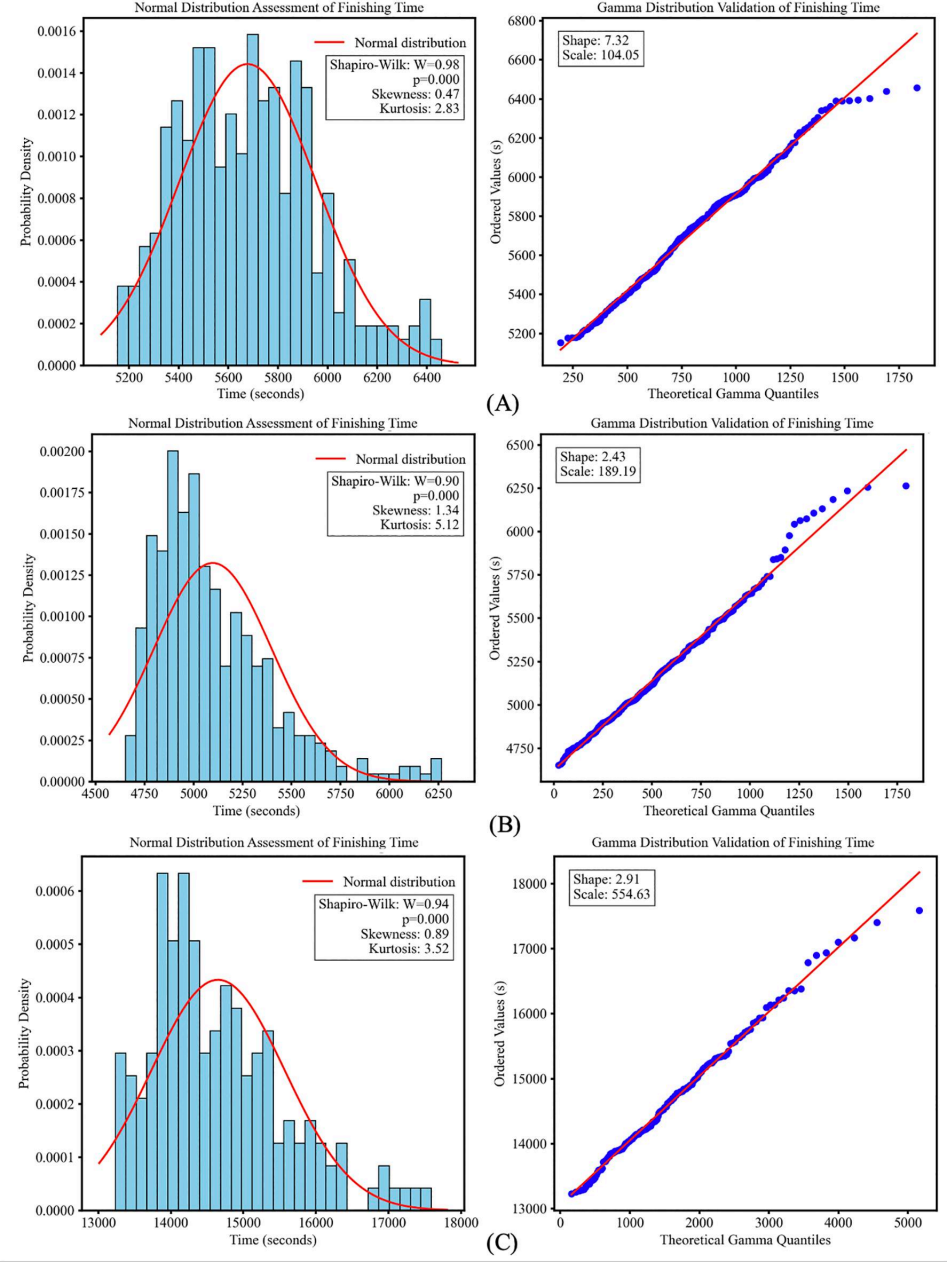
Finishing time distributions in racewalking. Left panels: histograms showing right-skewed distributions, deviating from the overlaid normal distribution curve (red line). Right panels: Q-Q plots validating the gamma distribution model for finishing times. Data points closely following the gamma reference line (red) support the gamma model. **(A)**: Women's 20 km; **(B)**: men's 20 km; **(C)**: men's 50 km.

Visual analysis of Q-Q plots (Figure 1) showed systematic deviations from the theoretical normal distribution quantiles while exhibiting closer proximity to the expected quantile pattern of a gamma distribution. Histograms consistently displayed the right-tailed asymmetry across all disciplines.

### 3.2 Correlation analysis

**Figure 2:**
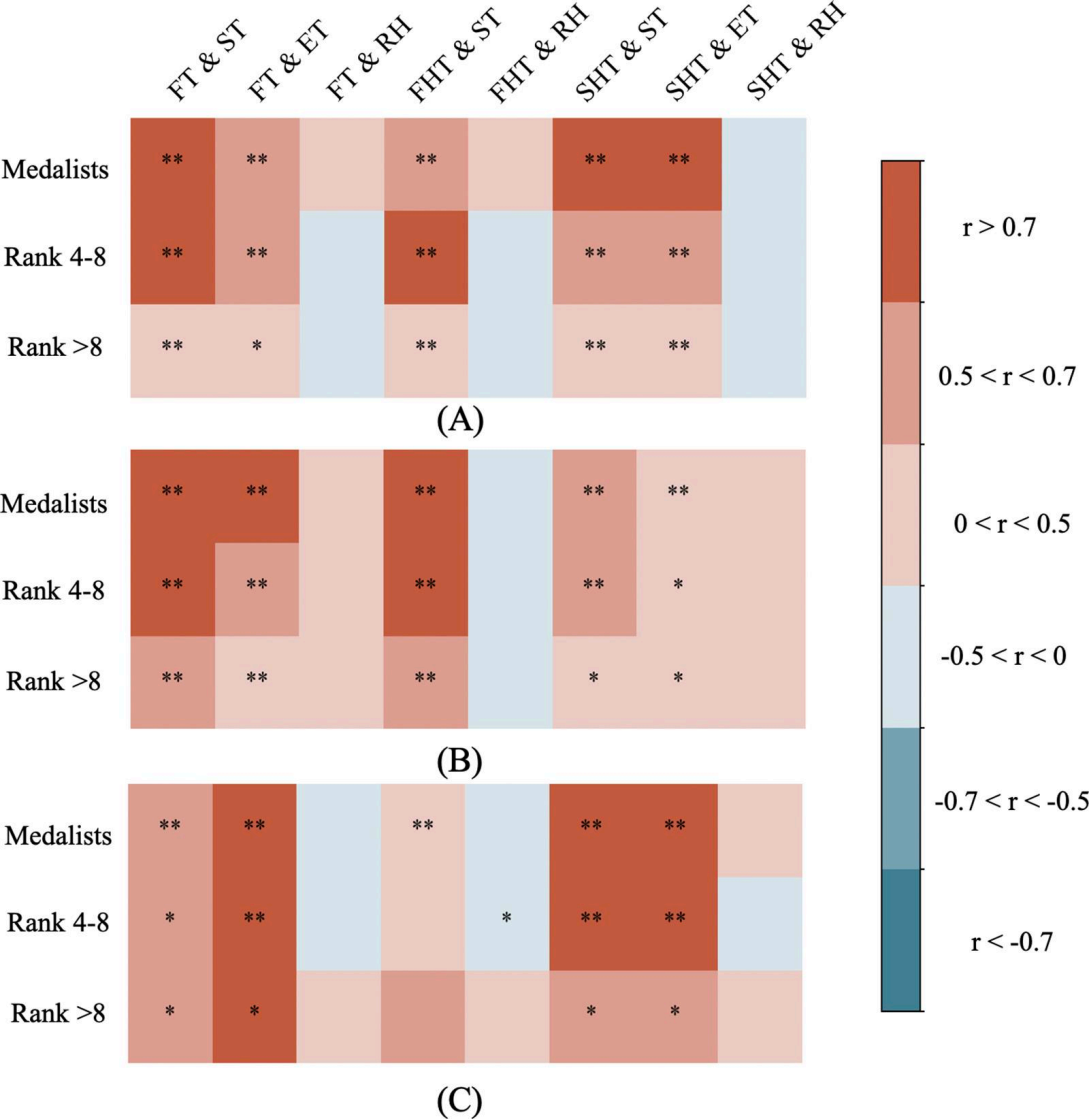
Correlation between weather conditions and racewalking results. Spearman correlation heat maps between weather variables (ST = starting temperature, ET = ending temperature, RH = relative humidity) and performance metrics (FT = finishing time, FHT = first half time, SHT = second half time) across performance levels. Map **(A)**: women's 20 km; Map **(B)**: men's 20 km; Map **(C)**: men's 50 km. Color intensity indicates correlation strength. *p < .05; **p < .001.

Significant associations were observed between ambient temperatures and racewalking performance, with distinct patterns across sex, performance levels, and race segments. For women's 20 km (Figure 2A), starting temperature (ST) and ending temperature (ET) showed moderate to strong correlations with finishing times for top8 athletes (*r* > 0.5). In contrast, athletes ranked below 8th exhibited only weak correlations (*r* = 0.40–0.42, *p* < .05).

In men's 20 km (Figure 2B), similar significant ambient temperature correlations were observed for top8 athletes, though ending temperature showed weaker associations with second half time (*r* = 0.44–0.45, *p* < .05). Athletes ranked below 8th demonstrated moderate correlations between starting temperature and both finishing and first half time (*r* = 0.53–0.61, *p* < .001) but weak correlations for the second half (*r* = 0.41–0.46, *p* < .05).

For men's 50 km (Figure 2C), no significant correlation was found between ambient temperatures and the first half time for athletes who ranked below 4th. However, top8 athletes developed moderate-to-strong temperature correlations during the second half (*r* = 0.63–0.76, *p* < .001). Relative humidity showed no significant association with 20 km performance, but weak correlations emerged for athletes ranked 4th–8th in the first half times of men's 50 km (*r* = −0.48, *p* < .05).

### 3.3 Regression modeling and visualization

The generalized linear models with Gamma distribution and log link function revealed associations between ambient temperature and entire performance in three disciplines (Table 2). The overall analysis demonstrated the strongest model fit for men's 50 km (Pseudo *R*^2^ = 0.29), followed closely by men's 20 km (*R*^2^ = 0.28) and women's 20 km (*R*^2^ = 0.19). Starting temperature showed significant positive correlations with finishing times across all disciplines, with men's 20 km displaying the highest sensitivity (+1.15%), followed by men's 50 km (+0.96%) and women's 20 km (+0.77%). Ending temperature associations were smaller in magnitude and exhibited directional differences among disciplines.

**Table 2:** Overall ambient temperatures effects on finishing time.

Disciplines	*N*	Mean finishing time	Pseudo *R*^2^	Starting temp.	Ending temp.
Women's 20 km	363	1:33:39	0.19	+0.77%(+43s)**	−0.36%(−20s)**
Men's 20 km	399	1:24:41	0.28	+1.15% (+58s)**	−0.66% (−33s)**
Men's 50 km	163	3:59:45	0.29	+0.96% (+140s)**	+0.28% (+41s)*

* *P* < .05.

** *P* < .001.

Stratified models improved explanatory power (Table 3), particularly for top8 athletes. In women's 20 km, top8 athletes showed greater temperature sensitivity (+0.64 to +0.67%) compared to lower ranked athletes (+0.42%), with ending temperature showing a significant negative association only between rank4–8 athletes (−0.26%). For male athletes, two distinct patterns emerged. First, regarding performance levels, athletes ranked below 8th showed greater temperature sensitivity (1.33%–3.14%) than rank4–8 groups (0.74%–1.57%) in both distances, with medalists in men's 20 km displaying no significance. Second, the associations between ambient temperature and racewalking performance showed opposite directions between distances: whereas men's 20 km performance decreased with starting temperature, the 50 km showed consistently positive starting temperature association across all rankings (−1.44% to −3.14%). Meanwhile, the ending temperature in the 50 km demonstrated increasing negative associations (+2.43% to +4.64%) on racewalking performance across performance levels. Combined temperature association differences across different performance levels were minor, ranging from 0.02% to 0.39%, with the smallest difference observed in women's 20 km.

**Table 3:** Stratified analysis of ambient temperature and racewalking performance.

Disciplines	Performance levels	Pseudo *R*^2^	Starting temp. (per1°C increase)	Ending temp. (per 1°C increase)	Combined (per 1°C increase)
Women's 20 km	Medalists	0.73	+0.64% (+35.76s)**	–	+0.43% (+23.86s)
	Rank 4–8	0.71	+0.67% (+37.38s)**	−0.26% (−14.45s)*	+0.41% (+22.84s)
	Rank >8	0.23	+0.42% (+23.82s)**	–	+0.45% (+25.48s)
Men's 20 km	Medalists	0.65	–	–	+0.64% (+32.58s)
	Rank 4–8	0.54	+0.74% (+37.55s)*	–	+0.51% (+25.88s)
	Rank >8	0.46	+1.33% (+67.71s)**	–	+0.85% (+43.36s)
Men's 50 km	Medalists	0.86	−1.44% (−206.12s)*	+2.43% (+349.47s)**	+0.96% (+138.34s)
	Rank 4–8	0.83	−1.57% (−226.06s)**	+2.54% (+365.70s)**	+0.93% (+133.90s)
	Rank >8	0.64	−3.14% (−452.04s)**	+4.64% (+667.15s)**	+1.35% (+194.15s)

* *P* < .05.

** *P* < .001.

Segment-based analysis (Table 4) showed stronger model explanatory power in the first half (Pseudo *R*^2^ = 0.30–0.74) than in the second half. In women's 20 km, explanatory power in the second half dropped to 0.11, with no significance. In contrast, men's racewalking maintained significant ambient temperature associations (*R*^2^ = 0.28 and 0.24, respectively). In men's 50 km, higher starting temperatures in the second half were associated with better performance of −3.08%/°C, while higher ending temperatures were associated with poorer performance of +4.27%/°C. In men's 20 km, the starting temperature in the second half remained significantly associated with performance negatively (+0.99%, *p* < .001), while the ending temperature showed no significance.

**Table 4:** Segment-based analysis of ambient temperatures and racewalking performance.

Disciplines	Segment	Pseudo *R*^2^	Starting temp. (per 1°C increase)	Ending temp. (per 1°C increase)
Women's 20 km	First half	0.30	+0.64% (+18s)**	−0.20% (−6s)*
	Second half	0.11	–	–
Men's 20 km	First half	0.52	+0.85% (+21s)**	–
	Second half	0.24	+0.99% (+26s)**	–
Men's 50 km	First half	0.74	−3.71% (−266s)**	+5.98% (+430s)**
	Second half	0.28	−3.08% (−222s)**	+4.27% (+308s)**

* *P* < .05.

** *P* < .001.

**Figure 3:**
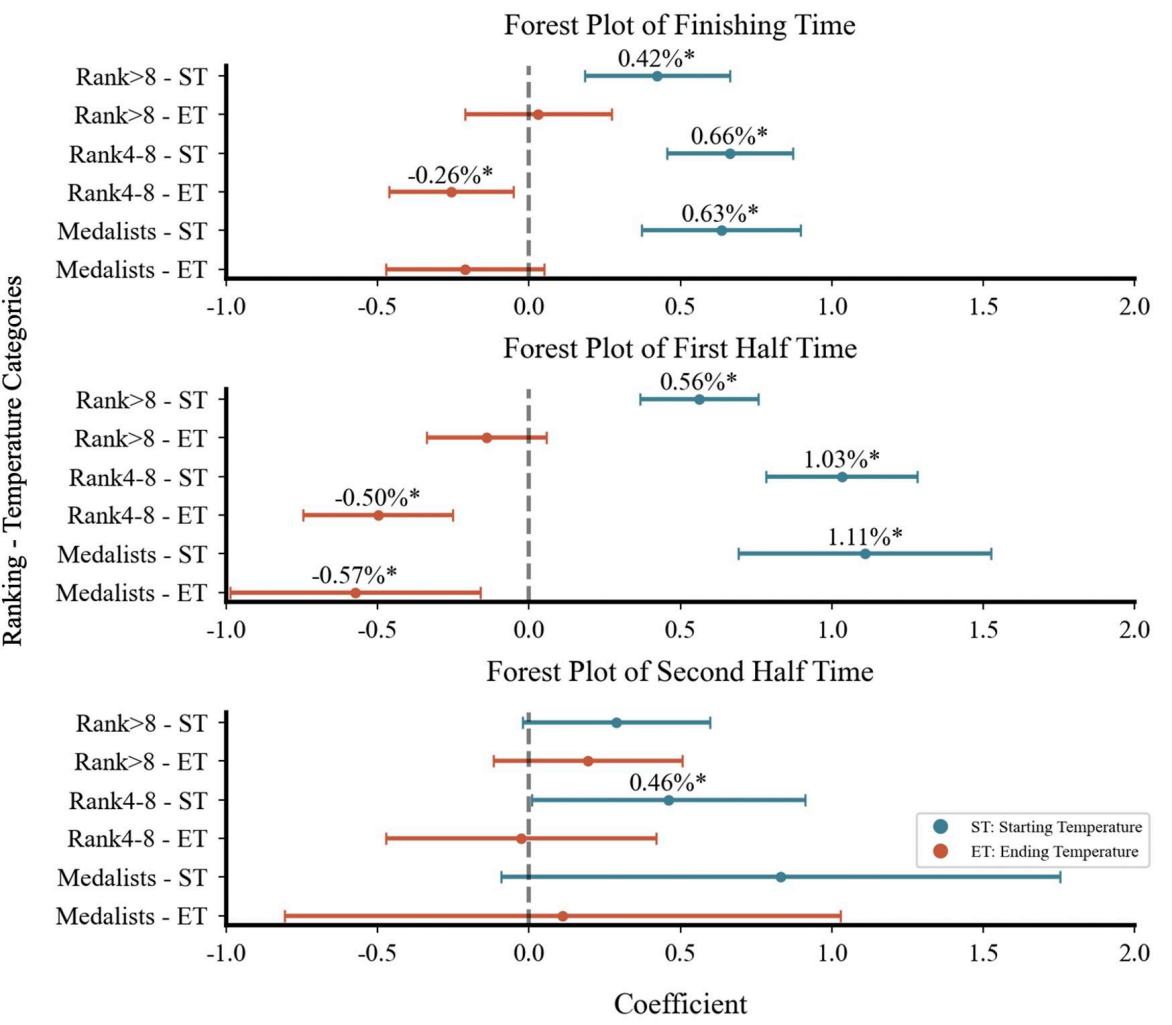
Forest plot of women's 20 km racewalking.

**Figure 4:**
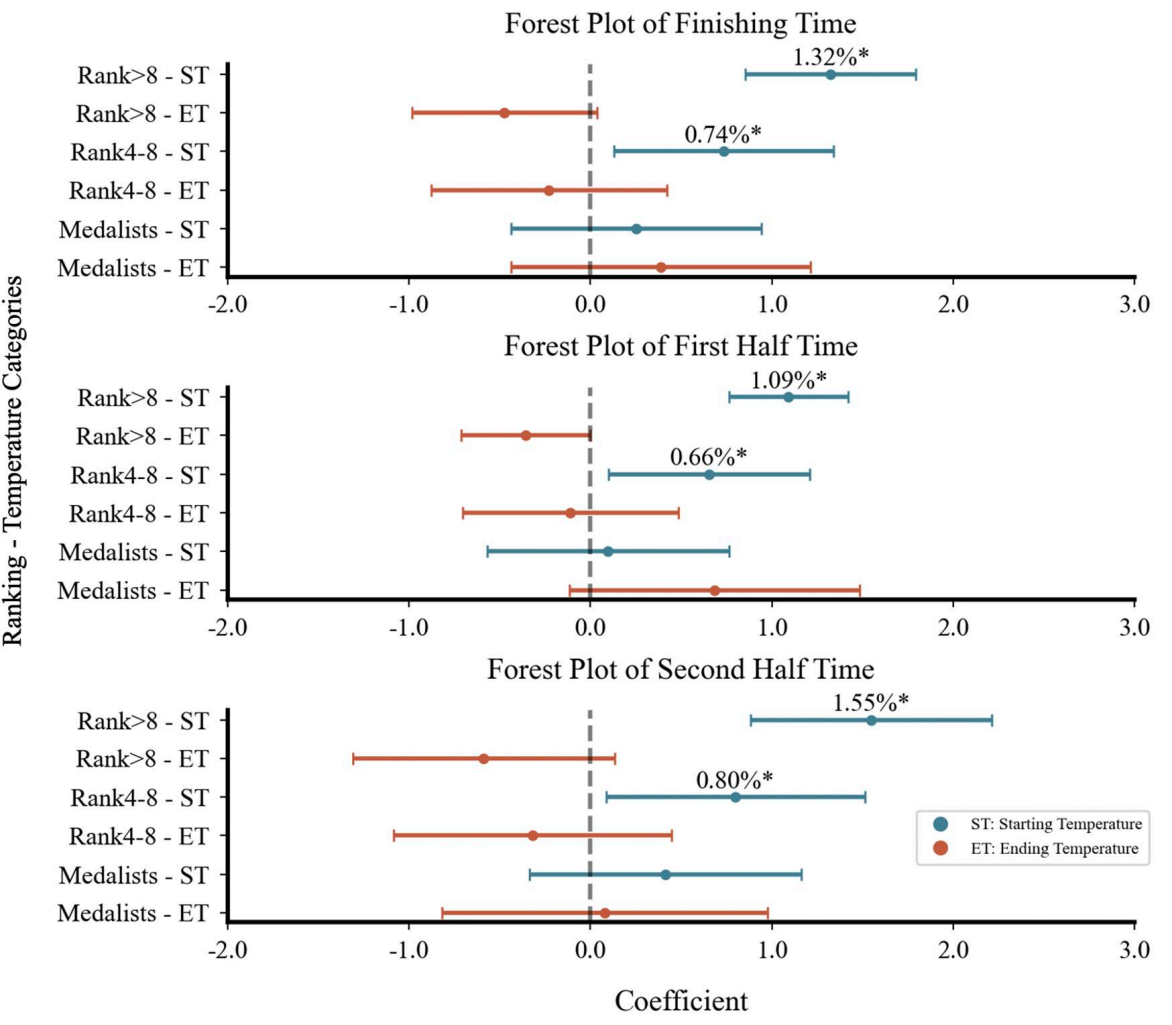
Forest plot of Men's 20 km racewalking.

**Figure 5:**
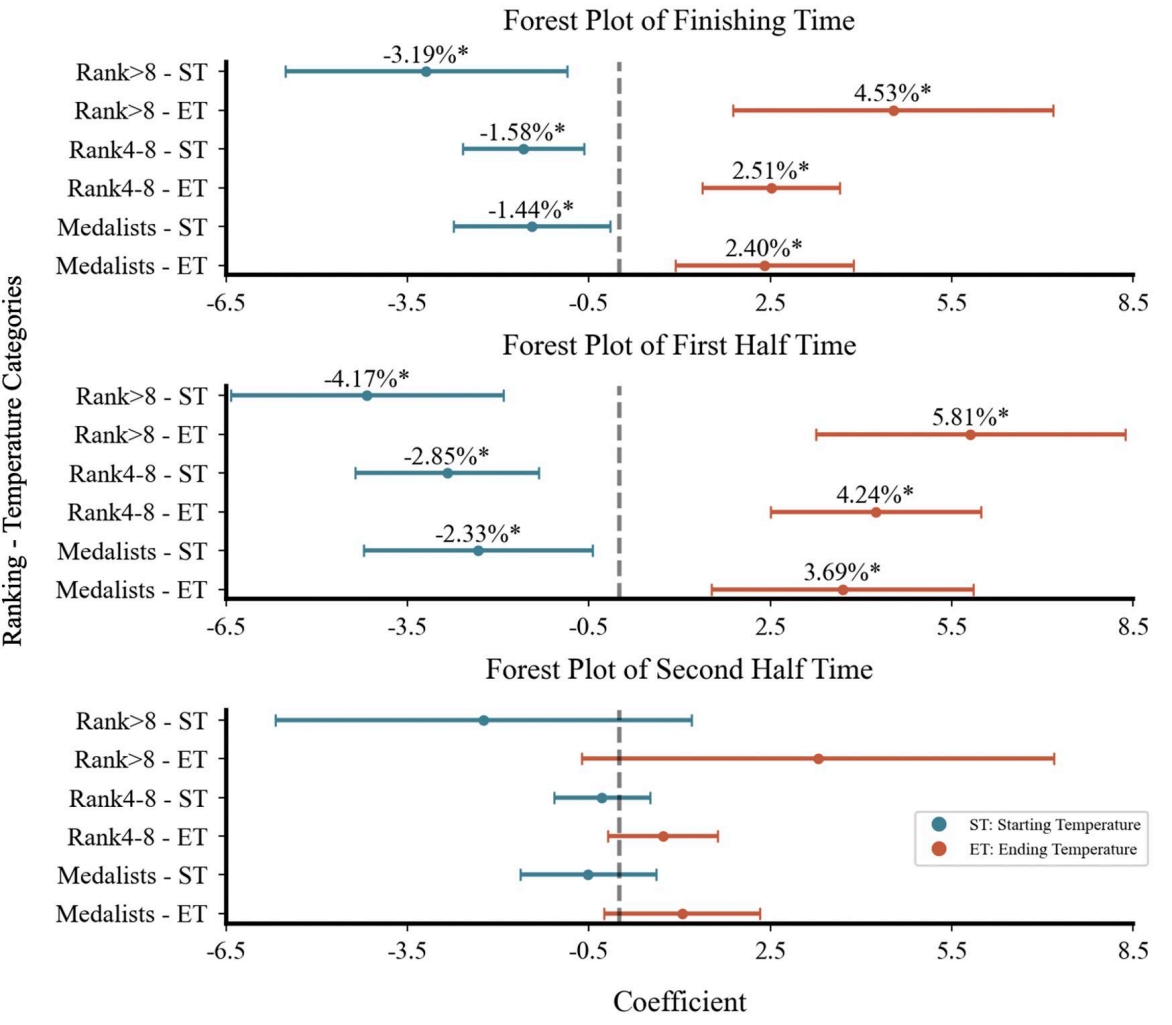
Forest plot of Men's 50 km racewalking. Figures 3–5 are forest plots illustrating the correlation between starting and ending temperatures and racewalking performance. Figure 3 shows data for women's 20 km, Figure 4 for men's 20 km, and Figure 5 for men's 50 km. Points represent estimated association magnitudes (*β* coefficients) with 95% confidence intervals. Values to the right of the null line (*β* > 0) indicate performance deterioration, while values to the left (*β* < 0) indicate improvement. *p < .05; **p < .001.

Forest plots (Figures 3–5) visually presented the magnitude, direction, and confidence intervals (CIs) of the associations between ambient temperature and racewalking performance. In women's 20 km (Figure 3), starting temperature showed an overlapping association on both finishing time and first half time. For the men's 20 km, the CIs for the associations between starting temperature and performance were more dispersed with less overlap. In the men's 50 km (Figure 5), both starting and ending temperatures influenced the finishing time and first half time, with differing directions of association and limited CI overlap.

## 4 Discussion

This study demonstrates significant associations between ambient temperature and elite racewalking performance, with stratified analyses revealing differences across sex, performance levels, and race segments. While Spearman's correlation indicates overall trends, GLMs isolate the independent associations of starting and ending temperatures, highlighting the value of multivariable modeling.

### 4.1 Sex differences

Female racewalking athletes show greater performance stability under high ambient temperatures, as evidenced by smaller association magnitudes and narrower confidence intervals in the regression models compared to males. This suggests that female athletes are less sensitive to elevated ambient temperatures.

Physiologically, female athletes typically benefit from a higher surface area-to-mass ratio, facilitating more efficient heat dissipation (23). They also tend to maintain lower heart rate, skin, and core temperatures in hot conditions, enhancing their thermoregulatory capacity relative to males (11, 23, 24). In contrast, male athletes generally exhibit weaker thermoregulatory responses and thus experience more pronounced performance variation under thermal stress.

Strategically, male athletes often adopt more aggressive pacing, especially in the early stages of the race, leading to faster heat accumulation and performance decline in high ambient temperatures (23–25). Female athletes, however, employ conservative pacing strategies (10, 12, 25–32), which moderates core temperature elevation and promotes consistent performance. These physiological and strategy differences may collectively explain the observed sex-based variation in thermal stress resilience among elite athletes.

### 4.2 Performance levels

Beyond the strategic implementation of pacing, it is crucial to consider the role of external thermal management strategies, which may also contribute to the performance differentials observed across various levels. Under high ambient temperature, athletes extensively employed cooling strategies to mitigate heat stress. Pre-cooling protocols included both internal and external methods, ranging from the application of ice vests and cold towels (33, 34) to more immersive techniques like a 30-minute cold water immersion (34). During competition, mid-cooling was even more widespread, with over 90% of athletes in one major championship employing such aids (33). These included water dousing of the head and face (33, 34), scheduled intake of ice-cold water or isotonic beverages at approximately 10-minute intervals (35), and the application of sensory agents such as menthol gel (34). It is plausible that higher-ranked athletes benefit from more sophisticated and effective cooling protocols and support systems than lower-ranked athletes (33). Therefore, the effective application of these external interventions, in conjunction with optimized pacing, likely forms a more comprehensive explanation for the enhanced thermoregulatory resilience seen in higher-ranked athletes.

In men's racewalking, except for the medalists in the 20 km, the starting temperature showed progressively stronger negative associations with performance as ranking levels declined. In the 50 km, all athletes demonstrated temperature sensitivity, with stronger associations observed in lower-ranked athletes. A previous study found that pacing strategies differ markedly among male athletes (25), with higher-ranked athletes maintaining higher average speeds and more stable pacing (36). Medalists in men's 20 km racewalking frequently start conservatively (37), pacing below their personal bests (38), enabling better thermal management. Consistent with these observations, our analysis revealed an association between elevated ambient temperatures and performance that appeared more pronounced among lower-ranked male athletes compared to higher-ranked athletes.

In contrast, in women's 20 km, the medalists and 4th–8th athletes exhibited greater sensitivity to ambient temperature variations compared to those ranked below 8th, indicating different stratified responses among elite athletes in heat adaptation. Although female athletes generally employ more consistent pacing strategies (23) and possess stronger thermoregulatory abilities (10, 12, 25–32), top8 athletes may experience greater relative slowing under high ambient temperature due to their initially faster pacing, as the steady deceleration among low-ranked athletes may obscure ambient temperature associations, whereas faster athletes exhibit more pronounced pacing declines under heat stress (39). Critically, the current study found that higher ending temperatures were associated with the first half performance deterioration in top8 athletes, further suggesting the potential dynamic, segment-based links to temperature that may underlie observed performance differences.

### 4.3 Race segments

Ambient temperature influenced performance differently across race segments. In the first half, the starting temperature showed significant associations with performance across most performance levels groups, excluding the medalists in the men's 20 km, while the ending temperature affected the first half times in all except the men's 20 km. In the men's 20 km, higher ambient temperatures were associated with performance impairment, whereas in the men's 50 km, a positive correlation with performance was observed. Female athletes exhibited generally low sensitivity to ambient temperature, though top8 athletes showed marginally stronger associations between ambient temperature and performance than those ranked below 8th. This may relate to their documented thermoregulatory characteristics: while possessing superior heat dissipation capacity (10, 12, 23, 25–32), female athletes may require extended exposure durations to fully engage heat acclimation processes (40, 41), leading to delayed performance stabilization in the second half. The demands of men's 50 km, particularly its prolonged distance, may result in different physiological patterns. The conservative early pacing required by technical regulations (42) in the men's 50 km effectively minimizes initial heat accumulation (38) despite prolonging exposure time during the first half due to reduced speed. This strategy facilitates progressive thermoregulatory stabilization, which combined with sustained steady pacing in later stages (42, 43) consequently reduces thermal strain despite the initial heat exposure, demonstrating how strategic pacing and long-distance characteristics collectively optimize thermoregulation.

In the second half, ambient temperature was associated with performance in the men's 20 km, while its association with performance had a diminished impact in the women's 20 km and men's 50 km. These patterns suggest that early thermoregulation capacity may determine the subsequent performance. Male athletes in the 20 km tend to adopt aggressive early pacing (13, 23, 24, 44), which may exceed their thermoregulatory limits, resulting in rapid core temperature rise, earlier onset of heat strain, and subsequent fatigue-induced slowdown in the second half (25, 45). Key performance differentials among top8 racewalking athletes often emerge in the second half (46), underscores the decisive role of pacing sustainability under heat stress. In contrast, the more even pacing strategies adopted by female 20 km and male 50 km athletes (23, 30, 42) likely facilitate better thermal regulation and performance resilience in later stages.

When examining finishing times, we observed consistent associations between first half times and ambient temperature across disciplines, suggesting that the first half may be more strongly linked to the entire performance. The second half generally showed weaker associations, with starting temperature associated with second half times only in men's 20 km racewalking. These patterns indicate that early-race thermal conditions may have a prolonged association with final results. Effective heat management in the first half could be particularly important for top8 qualification, while second half performance variability may contribute more to final ranking differences (46).

## 5 Conclusions

This study identified significant associations between ambient temperature and elite racewalking performance across sex, performance levels, and race segments. The findings offer observational evidence that complements existing experimental research on thermal stress in endurance sports.

Female athletes generally show greater stability under high ambient temperatures than males, indicating better thermoregulation and conservative pacing strategies. Males show greater performance variability, which may be attributed to early aggressive pacing and less efficient heat dissipation. Stratified analysis indicated that female top8 and male athletes who ranked below 4th were more sensitive to ambient temperature fluctuations, highlighting the need for targeted strategies for these groups, such as the personalized application of cooling aids (e.g., pre-race ice vests and mid-race water dousing or intake of ice-cold beverages). Segment-based analysis further indicated that the starting temperature was associated with overall performance, particularly during the first half of the race, emphasizing the critical role of early thermal management.

Building on these findings, we propose several practical applications. The strong association between starting temperature and the first half performance suggests that segment-specific pacing should be a primary focus. To avoid substantial performance degradation in the second half, athletes, especially male, should consider adopting a more conservative initial pacing strategy. Moreover, given the observed variation in temperature sensitivity, individualized race strategies are essential. For instance, the top8 female athletes and male athletes who ranked below 4th, groups that showed higher sensitivity, may need to extend the conservative initial strategy into a more comprehensive and meticulously planned pacing strategy for the entire race. In addition to pacing adjustments, a comprehensive thermal management plan should incorporate proactive cooling interventions. Pre-cooling methods and strategies can be instrumental in managing core body temperature, particularly before and during the first half of the race. Therefore, a holistic approach that integrates pacing, hydration, and cooling protocols is essential for enhancing thermal resilience and optimizing performance for elite athletes in racewalking.

## 6 Limitation

While this study offers valuable insights, several limitations should be noted. First, the research focused on major international events to establish a homogeneous cohort of elite athletes. While our statistical approach effectively addressed issues arising from unequal group sizes, the findings are based on a limited number of events. Therefore, future research with larger cohorts is warranted to confirm the broader applicability and stability of these observed patterns. Second, our analysis was constrained by the inability to incorporate several key meteorological variables—notably wind speed, solar radiation, and comprehensive thermal indices—due to a lack of consistent official data. The exclusion of these factors limits the interpretability of our findings, as the effect of an included variable like relative humidity is likely moderated by its interaction with these unmeasured conditions. Consequently, the non-significant finding for relative humidity should be interpreted with caution. This is highlighted by the 2019 Doha World Championships: by holding the race at midnight to eliminate solar radiation, the event demonstrated that the thermal environment had a severe impact on performance, underscoring the need for a comprehensive, multi-variable model to fully account for the interplay of all environmental factors.

## Data Availability

The data analyzed in this study was obtained from publicly available third-party sources: the official websites of World Athletics, the International Olympic Committee (IOC), and WeatherSpark. This data is publicly available and can be accessed directly from these sources.

## Supplementary material

Supplementary Figure S1 Outlier detection using box plots. Box plots of finishing times for the **(A)** Women's 20 km, **(B)** Men's 20 km, and **(C)** Men's 50 km disciplines. The central box for each plot represents the interquartile range (IQR), with the vertical line inside marking the median. The whiskers extend to the furthest data points within 1.5 times the IQR from the first and third quartiles. Individual points plotted beyond the whiskers are potential outliers identified by the 1.5 × IQR rule.
