# The association between ambient temperature and elite racewalking performance in the olympics and world championships

**DOI:** 10.3389/fspor.2025.1749487

**Published:** 2025-12-08

**Authors:** Xiangning Zhang, Dandan Cui, Zili Jiang, Wenchao Yang

**Affiliations:** 1 Institute of Artificial Intelligence in Sports, Capital University of Physical Education and Sports, Beijing, China; 2 China Institute of Sport Science, Beijing, China; 3 Chinese Athletics Association, Beijing, China

**Keywords:** race segments, performance levels, elite athlete, racewalking, athletics

There was a mistake in Figure 2 as published. An incorrect image was submitted during the production process. The corrected Figure 2 appears below.

**Figure 2:**
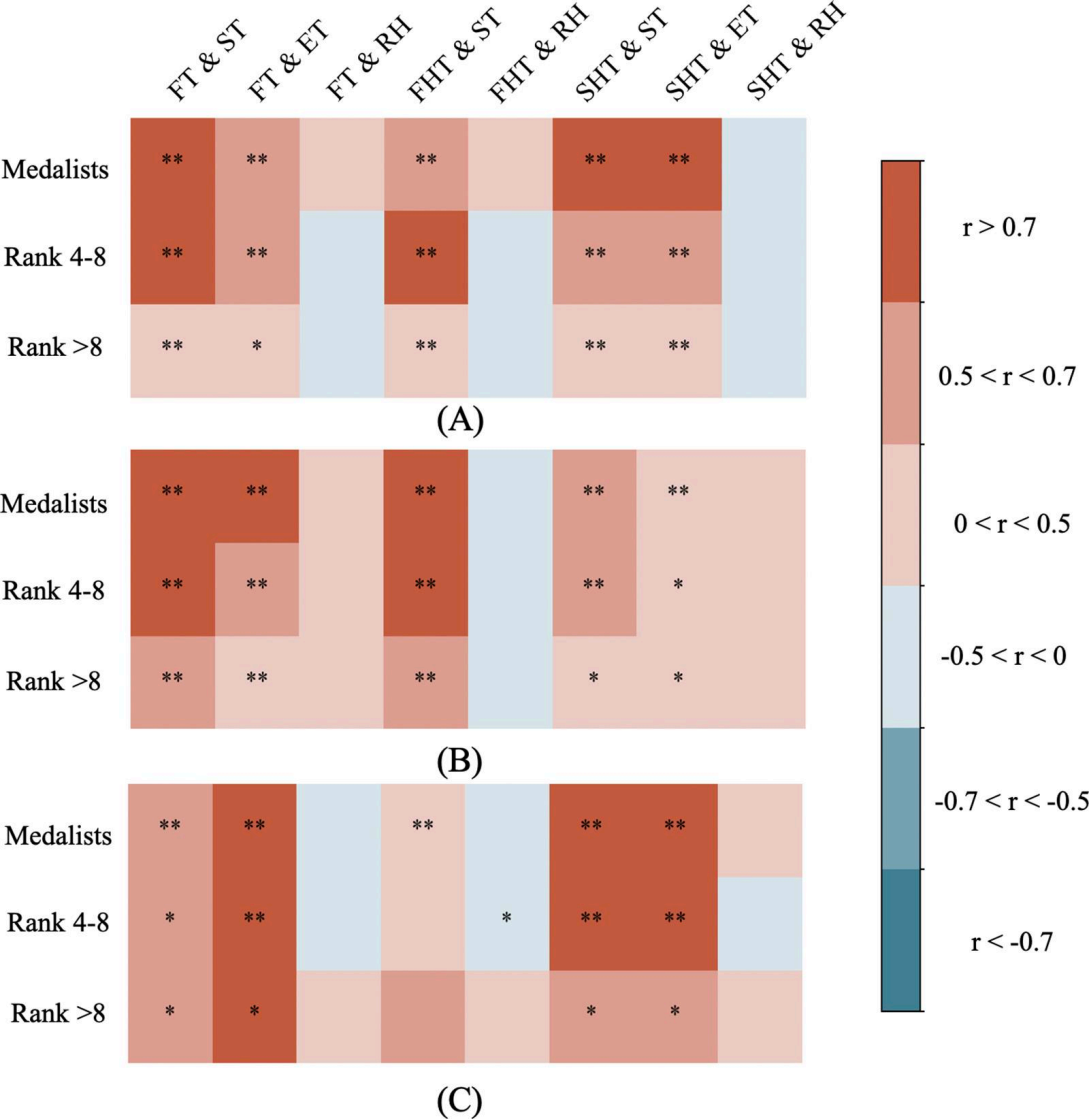


The original version of this article has been updated.

