# Supplementary figures and images for: The association between ambient temperature and elite racewalking performance in the olympics and world championships
Source: Front Sports Act Living. 2025 Nov 7;7:1681100. doi: 10.3389/fspor.2025.1681100 (PMC12634663; doi:10.3389/fspor.2025.1681100)

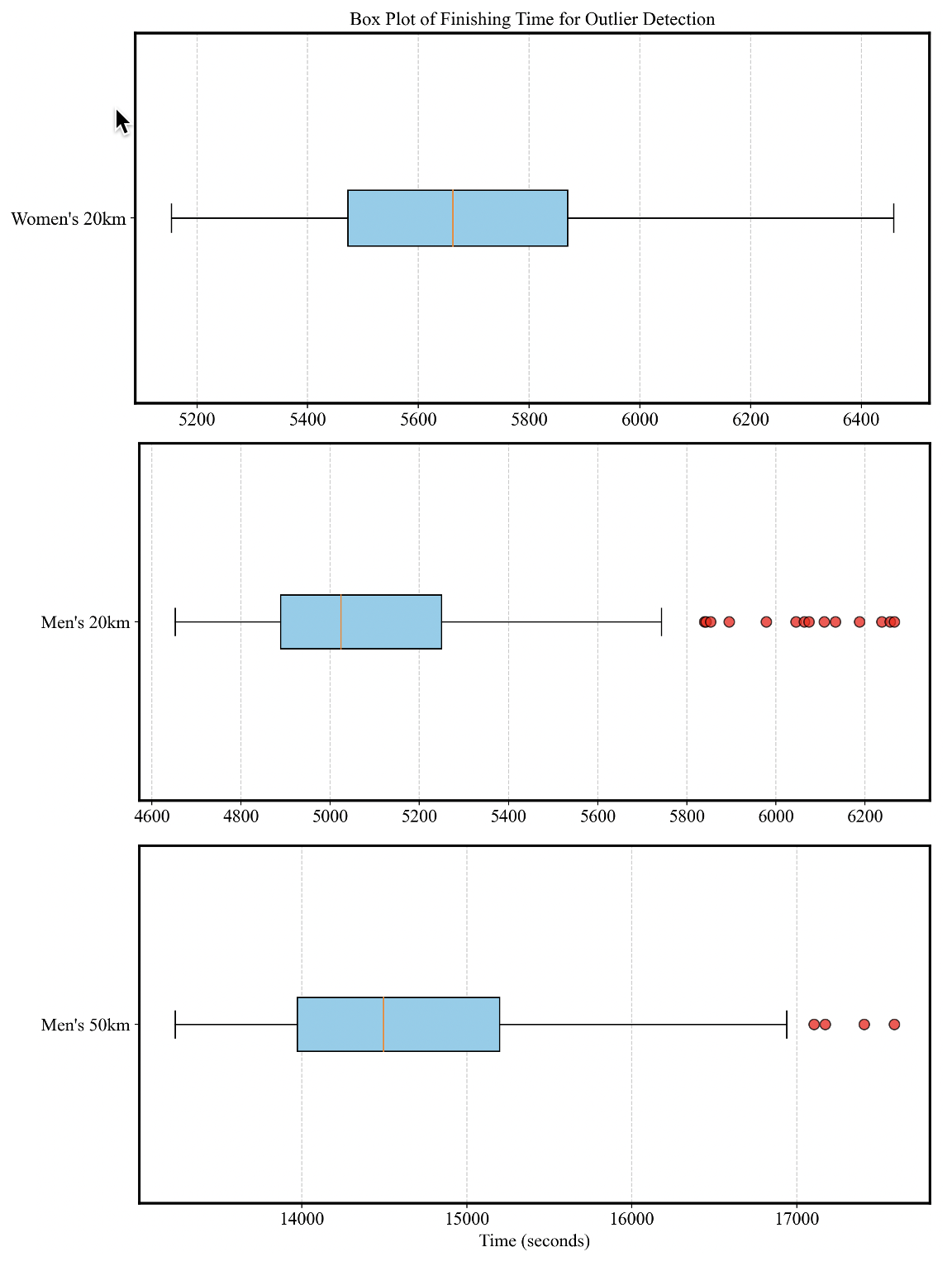

Supplement: Supplementary Figure S1 — Outlier detection using box plots. Box plots of finishing times for the (A) Women's 20 km, (B) Men's 20 km, and (C) Men's 50 km disciplines. The central box for each plot represents the interquartile range (IQR), with the vertical line inside marking the median. The whiskers extend to the furthest data points within 1.5 times the IQR from the first and third quartiles. Individual points plotted beyond the whiskers are potential outliers identified by the 1.5 × IQR rule. [file Image1.tiff]
